# Retrocorneal Scleral Patch Supported Glue: A Technique for Management of Corneal Perforation and Corneoscleral Melt following Pterygium Surgery

**DOI:** 10.18502/jovr.v18i1.12732

**Published:** 2023-02-21

**Authors:** Ashok Sharma, Rajan Sharma, Verinder S. Nirankari

**Affiliations:** ^1^Ashok Sharma, MS. Cornea Centre, SCO 2463 - 2464, Sector 22C, Chandigarh 160022, India

**Keywords:** Corneal Perforation, Cyanoacrylate Tissue Adhesive, Pterygium Surgery, Scleral Necrosis, Scleral Patch

## Abstract

**Purpose:**

To describe a new method of treatment of corneal perforation with extensive corneoscleral melt.

**Case Report:**

A 42-year-old man presented with moderate-sized (3.5 mm) corneal perforation with extensive corneo-limbo-scleral ulceration following bare sclera excision of pterygium. No prior use of antimetabolites or postoperative beta radiation noted. We considered retrocorneal sclera patch supported cyanoacrylate application. The sclera was thinned to one-third thickness and a patch (4.5 × 4.5 mm) was punched. The sclera patch was placed on the iris, behind the corneal perforation, adequately covering it from inside. A minimal amount of adhesive was applied on the retrocorneal sclera patch and margin of corneal perforation. The ulcerating sclera was covered with double layered amniotic membrane. Topical antibiotic, steroid, and cycloplegic drops were instilled thrice daily. Corneal perforation healed and no recurrence occurred during the 18 months' follow-up.

**Conclusion:**

Retrocorneal scleral patch supported cyanoacrylate is effective for corneal perforation with corneo-scleral melt.

##  INTRODUCTION

Pterygium excision with conjunctival autograft, pterygium excision with amniotic membrane graft, and pterygium excision with combined conjunctival autograft and amniotic membrane graft have been advocated for primary or recurrent pterygium.^[1]^ The bare sclera technique, although technically simple and easy, is associated with a high incidence of recurrence (62%) and is no longer favored.^[2]^ Pterygium excision with conjunctival autograft is the preferred treatment modality for primary pterygium.

Scleral necrosis has been reported following the bare sclera excision technique and the use of beta radiation and antimetabolites (thiotepa and mitomycin C) in the postoperative period.^[2,3]^ Cases of scleral necrosis following the bare sclera technique without the use of beta radiation and antimetabolites have also been reported.^[3]^ Corneal ulceration may be treated with amniotic membrane graft, deep anterior lamellar keratoplasty, or therapeutic penetrating keratoplasty.^[4]^ Scleral ulceration may be treated with amniotic membrane graft, conjunctival autograft, Tenon's capsule graft, and scleral graft.^[5]^ Moderate corneal perforation, extensive corneo-limbo-scleral melt is an extremely rare complication of pterygium surgery and poses a management challenge. In the presence of the extensive corneo-limbo-scleral ulceration, deep anterior lamellar keratoplasty or therapeutic penetrating keratoplasty could not be considered. Application of cyanoacrylate tissue adhesive alone also was not feasible, as perforation was large. We report such a case successfully managed with retrocorneal sclera patch augmented cyanoacrylate tissue adhesive application.

##  CASE REPORT

**Figure 1:**
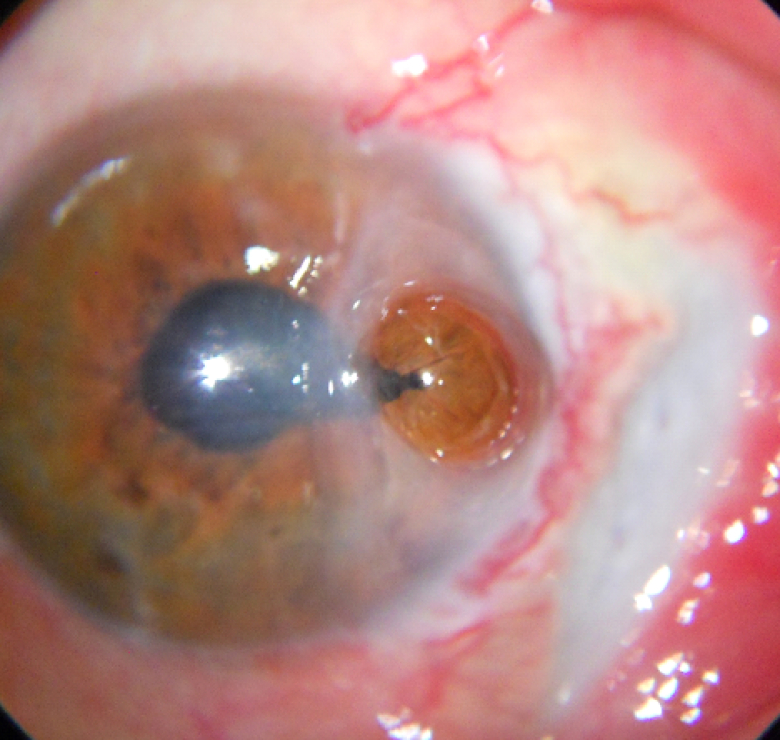
Corneal perforation (3.5 mm) with iris presenting at the site with extensive corneo-limbo-scleral melt.

A 42-year-old man presented with persistent pain, redness, watering and diminution of vision in the right eye for three weeks. Referring ophthalmologist performed pterygium surgery using bare sclera technique and patient was fine for four weeks and then developed symptoms. The referring ophthalmologist treated the patient with topical antibiotic and steroid for three weeks. Observing no response, the patient was referred to*.* No antimetabolites or postoperative beta radiation were used in this case. The operating surgeon had used heat cautery on the sclera. Patient did not reveal any history suggestive of systemic autoimmune disorder. At the time of presentation, his visual acuity was 4/60 in the right eye and 6/6 in the left eye. Intraocular pressure in the left eye was 16 mmHg. Slit-lamp biomicroscopy revealed large area of corneal perforation (3.5 × 3.5 mm) and extensive corneo-limbo-scleral necrosis [Figure 1]. The left eye did not reveal any abnormality.

The material obtained on corneal scrapings were subjected to the detailed microbiological tests. Direct microscopy and cultures of the scraping material did not reveal any bacterial or fungal pathogen. Hematological investigations including complete blood count, erythrocyte sedimentation rate, rheumatoid factor, anti-nuclear antibody, ant-DNA antibodies, liver function tests, renal function tests, C-reactive protein and antineutrophil cytoplasmic antibodies were normal. Serology markers of human immunodeficiency virus 1 and 2, varicella-zoster virus, hepatitis B surface antigen, hepatitis C and venereal disease research laboratory test were non-reactive. Chest radiography and the Montoux test were normal.

**Figure 2:**
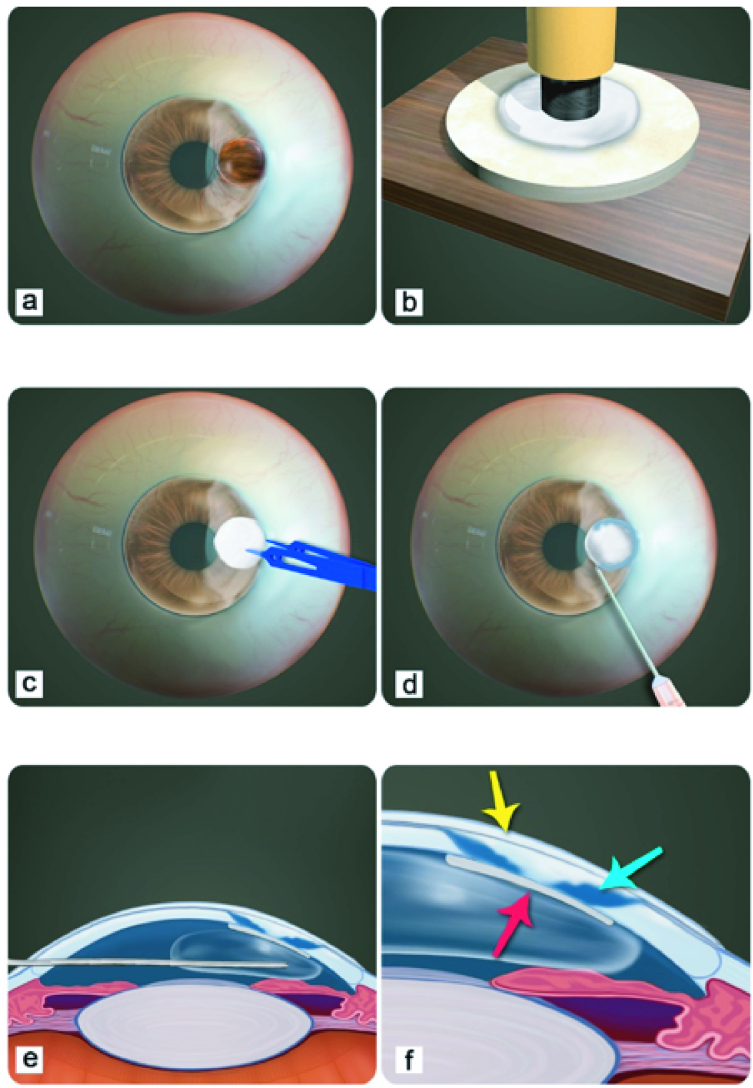
Diagramatic presentation of retrocorneal sclera patch supported cyanoacrylate tissue adhesive application: (a) Corneal perforation (3.5 × 3.5 mm), corneal, limbal and sclera melt; (b) Punching of sclera patch (4.5 × 4.5 mm) using a skin bopsy punch; (c) Placing the thinned sclera patch behind the corneal perforation; (d) Application of minimum quantity of cyanoacrylate tissue adhesive at the margin of perforation on the sclera patch; (e) Injecting an air bubble in the anterior chamber; (f) At completion of surgical procedure, retrocorneal sclera patch (red arrow), cyanoacrlate tissue adhesive (blue arrow), and bandage contact lens (yellow arrow).

**Figure 3:**
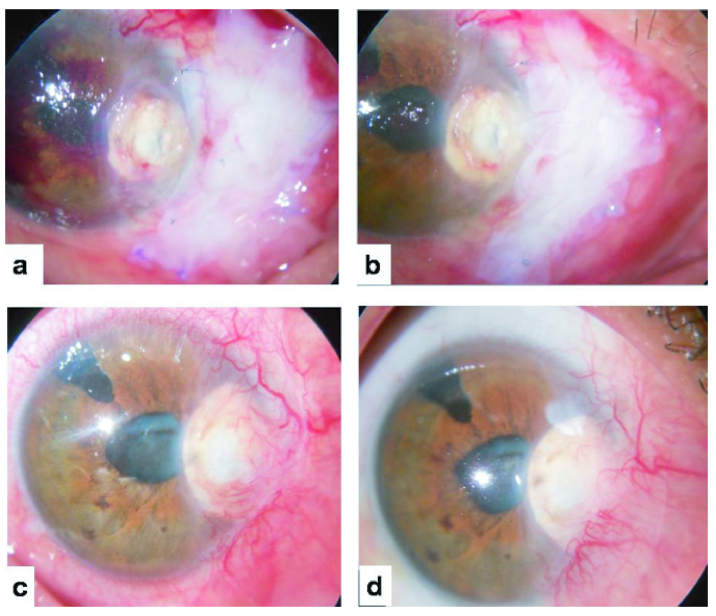
(a) Sealed corneal perforation 72 hr after surgery (scleral patch, CTA plug, and double layered AMG in place). (b) Corneal perforation sealed and corneo-limbo-scleral necrosis at healing stage at two-weeks' follow-up. (c) Healed corneal perforation (sclera patch in place) and pseudo-pterygium at 12 weeks' follow-up. (d) Healed corneal perforation (sclera patch in place) and pseudo-pterygium at 18 months' follow-up.

### Surgical Technique

Retrocorneal scleral patch augmented cyanoacrylate tissue adhesive application was done under peribulbar anesthesia, in the operating room using surgical microscope. The right eye was prepared and draped aseptically. The ocular surface was exposed using Barraquer eye speculum. The area of corneo-limbo-scleral ulceration was cleaned and the debris was removed. The size of the corneal perforation was measured with Castroviejo calipers. The corneal perforation measured 3.5 × 3.5 mm [Figure 2a]. The donor sclera was obtained from eyes not suitable for optical PKP. Donor sclera obtained from the local eye bank and stored in pure glycerin was taken out and placed in the normal saline for 15 min. The sclera was placed in the solution containing amikacin sulphate 2% (Alfakim 500 mg/2 ml Ranbaxy India) and vancomycin hydrochloride 5% (Vanking 500 mg/vial Neon Laboratories India) for 5 min. The sclera was thinned to one-third of its thickness and a patch (4.5 × 4.5 mm) was punched out with skin biopsy punch [Figure 2b]. The scleral patch was thinned to one-third of its thickness with crescent knife. The adhesions between the iris and the margin of corneal perforation were lysed. A small iridotomy was performed. The scleral patch was placed on the iris, beneath the corneal perforation [Figure 2c]. The scleral patch and the surrounding cornea surface were dried with Weck-cel ophthalmic sponge. Isoamyl 2-cyanoacrylate (Amcrylate; Concord Drugs Ltd, Hayathnagar, Andhara Pradesh, India) was drawn into a 2 ml disposable syringe with a 26 gauge needle. The minimal amount of cyanoacrylate tissue adhesive (Amcrylate; Concord Drugs Ltd, Hayathnagar, Andhara Pradesh India) was applied on the retrocorneal sclera patch and margin of the corneal perforation [Figure 2d]. The cyanoacrylate tissue adhesive was allowed to polymerize [Figure 2e]. An air bubble was placed in the anterior chamber. The ulcerating limbus and the sclera were covered with double-layered amniotic membrane graft. Amniotic membrane graft was sutured with Vicryl 8 `0' suture. A bandage contact lens was placed [Figure 2f].

The patient was put on topical moxifloxacin 0.5% (Vigamox; Alcon Laboratories, Inc., Fort Worth, TX) four times a day, atropine sulfate 1% (Atropine; Jawa Pvt. Ltd., Gurgaon, Haryana, India) three times a day, and carboxymethylcellulose 1% (Refresh Liquigel; Allergan, Pithampur, Madhya Pradesh, India) four times a day. After 72 hr, topical prednisolone 1% (Allergan Ltd, West-port, Co, Mayo, Ireland) four times a day was added. The patient was given intravenous methylprednisolone (1 gr) daily for three days followed by oral prednisolone 60 mg daily for two weeks and tapered over six weeks.

At 72 hr, the scleral patch, cyanoacrylate tissue adhesive plug, and double layered amniotic membrane graft were in place [Figure 3a]. At two weeks, corneal perforation was sealed and scleral necrosis started healing [Figure 3b]. At eight weeks' follow-up, cyanoacrylate tissue adhesive plug became lose and the scleral patch developed vascularization. The cyanoacrylate tissue adhesive plug was carefully separated from the underlying sclera patch and removed. At 12 weeks, the corneal perforation healed and the patient developed pseudo-pterygium. At 20 weeks, patient was diagnosed as suffering from intumescent cataract. Cataract surgery was performed. He was advised excision of the pseudo-pterygium but was reluctant [Figure 3c]. During the 18 months' follow-up, patient did not develop any recurrence, the scleral patch remained in place and the patient had pseudo-pterygium [Figure 3d].

##  DISCUSSION 

Retrocorneal scleral patch supported cyanoacrylate tissue adhesive application healed the corneal perforation and the amniotic membrane graft healed the corneo-limbo-scleral melt following pterygium surgery. Cyanoacrylate tissue adhesive is the gold standard treatment for corneal perforation.^[6]^ Cyanoacrylate tissue adhesive application used alone was not feasible because of the size (3.5 mm) of the perforation and also due to the risk of its inadvertent access into the anterior chamber.^[7]^ Due to extensive corneo-scleral ulceration around the corneal perforation, we did not consider scleral patch augmented cyanoacrylate tissue adhesive application technique.^[8]^ In this technique, the partial thickness scleral patch is placed on the perforation anteriorly, and cyanoacrylate tissue adhesive is applied on the margin and surface of the scleral patch.^[8]^ In another technique for moderate corneal perforations due to rheumatoid arthritis, a partial thickness scleral patch was placed in the intracorneal lamellar pocket and then cyanoacrylate tissue adhesive was applied.^[9]^ Since our patient had extensive corneal and sclera melt surrounding the corneal perforation, both these techniques could not be executed. The only option left to the authors was to place a sclera patch on the iris, behind the corneal perforation and then apply the cyanoacrylate tissue adhesive to seal the perforation. To the best of authors' knowledge, this technique has not been reported in literature. Amniotic membrane,^[10]^ Tenon's capsule,^[11]^ and cornea have also been used as tissue scaffolds to support cyanoacrylate tissue adhesive in treatment of moderate corneal perforations.

Bare-sclera technique of pterygium excision is not a favored treatment option.^[2]^ Most corneal surgeons prefer pterygium excision with conjunctival auto graft (CAG for both primary and recurrent pterygium.^[1]^ Bare sclera technique has high recurrence rate (60–80%).^[2]^ Bare sclera was previously combined with either post-op beta radiation or anti-metabolites (thiotepa or mitomycin-c). However, the use of both postoperative beta radiation and thiotepa/mitomycin-C has been reported to cause scleral necrosis.^[2]^ In most cases of scleral necrosis, mitomycin has been used several times more than the recommended concentration and dosage.^[1]^ These agents are used to reduce the recurrence of pterygium excision. The underlying pathogenesis of sclera melt include obliterative endarteritis and inhibition of mitosis in capillary endothelium. Therefore, the use of mitomycin with bare sclera technique is not advocated. Low-dose mitomycin-C associated with pterygium excision and limbal conjunctival grafts have been found to be successful.^[1]^ However, scleral necrosis and corneal melt have been reported following bare sclera technique in the absence of the use of postoperative beta radiation or anti-metabolite.^[3]^ In our patient, the primary operating surgeon did not use any postoperative beta radiation or anti-metabolite. Several ocular and systemic comorbidities can also predispose an inclination to sclera/cornea melt. Our patient did not have any collagen vascular disorder including rheumatoid arthritis or Wegner's granulomatosis.

To speculate the etiopathogenesis in our patient is difficult. Underlying etiopathogenesis may be infection, ischemia, or inflammation. Infective etiology was ruled out as all microbiological tests were negative. Primary ischemic etiology was unlikely as the patient had not received intra/postoperative mitomycin C. Light cautery application to bleeding vessels in the sclera bed could be held responsible for severe ischemia and scleral melt. It appears that bare sclera technique causing exposure of sclera was responsible for inducing inflammation that resulted in ischemia and ulceration. Clinical presentation and signs in our case simulate surgery-induced necrosis of the sclera.

In our patient, in addition to retrocorneal sclera patch augmented cyanoacrylate tissue adhesive application, double layered amniotic membrane graft was applied. Amniotic membrane graft coupled with intravenous methylprednisolone and later oral prednisolone decreased inflammation, arrested corneal scleral ulceration, promoted healing and epithelization. Patients not responding to corticosteroids or developing complications of oral steroids may need immunosuppressive agents.

In summary, this case report demonstrates that retrocorneal sclera patch augmented cyanoacrylate tissue adhesive healed the moderate sized (3.5 mm) corneal perforation in association with extensive corneo-limbo-scleral melt following pterygium surgery.

##  Financial Support and Sponsorship

None.

##  Conflicts of Interest

There are no conflicts of interest.
